# A Depth-Aware HGNN Method and Its Application in Anomaly Detection and Correction of Sparse Ocean Sensor Data

**DOI:** 10.3390/s26051537

**Published:** 2026-02-28

**Authors:** Zongxun Han, Xiang Gao, Zhengbao Li, Yugang Ren, Xianpeng Shi

**Affiliations:** 1 National Deep Sea Center, Qingdao 266237, China; hanzongxun_0820@163.com (Z.H.); ytrenyugang@163.com (Y.R.); xpsh@ndsc.org.cn (X.S.); 2 College of Ocean Science and Engineering, Shandong University of Science and Technology, Qingdao 266590, China; lizhengbao@sdust.edu.cn

**Keywords:** sparse ocean sensor data, anomaly detection and correction, heterogeneous graph neural network, depth-aware graph construction, DeepData database

## Abstract

In the field of ocean observation, we often face the challenge of the contradiction between the vast ocean environment and limited ocean sensor observations, resulting in significant sparsity in the acquired ocean sensor data. This sparse ocean sensor data typically exhibits characteristics such as discrete spatial distribution, discontinuous observation time, and vertical stratification with water depth variations. Current methods primarily employ rule-based quality control, time series modeling, or traditional graph neural networks for processing. This paper addresses the characteristics of sparse ocean sensor data, building upon these methods by further utilizing topological correlation and hierarchical feature modeling on a topological basis. It proposes a depth-aware heterogeneous spatiotemporal graph neural network (DAHSGNN) to achieve efficient anomaly detection and data correction for this type of data. DAHSGNN integrates discrete observation data along the depth axis using a local graph construction method. It employs hierarchical feature engineering to characterize the vertical stratification of the ocean. A Gaussian Hidden Markov Model is used to segment the water layers, and intra- and inter-layer trend features are extracted using a water layer probability-guided Transformer encoder. Then, a bidirectional long short-term memory deep sequence encoder captures the local dynamic context, thereby achieving fine-grained modeling of the ocean’s vertical stratification features. Finally, a heterogeneous graph autoencoder is used to reconstruct the site-level data distribution. Experiments were conducted using multiple environmental variables from the International Seabed Authority (ISA) DeepData database. Results show that DAHSGNN exhibits good cross-variable generalization ability, achieves higher reconstruction accuracy than baseline methods, and significantly improves anomaly detection performance.

## 1. Introduction

Approximately 71% of the Earth’s surface is covered by seawater, with about two-thirds of the ocean area located outside national jurisdiction. High-precision monitoring of this vast and remote ocean environment is one of the key challenges for global ocean scientific research and resource management. In some remote sea areas, the deployment density of Argo floats is insufficient, and monitoring of the deep-sea environment relies primarily on shipborne observations. Ocean sensor data is acquired through multi-parameter in situ sensors mounted on shipborne profilers, encompassing core raw measurement data such as depth and environmental parameters, as well as auxiliary information such as latitude, longitude, and time [1]. The DeepData database, established by the International Seabed Authority (ISA), is one of the most important deep-sea environmental databases globally. Its core data originates from relevant environmental and resource data obtained through shipborne observations of the International Seabed Area (The Area) [2], providing a crucial data foundation for global deep-sea environmental baseline assessment and continuous monitoring. Shipborne observation data is used to calibrate and cross-validate satellite inversion, the Argo profile and AUV/ROV observation data, thereby improving the accuracy of data assimilation and model-driven reconstruction. Shipborne surveys can efficiently provide cross-regional and cross-seasonal environmental samples, providing real-world data for deep-sea ecological baseline assessment, mining environmental impact assessment, and post-event monitoring and early warning. However, compared with nearshore observation stations or remote sensing observations, shipborne surveys are limited by a wide monitoring range and limited ship voyages, resulting in a lower spatial coverage density and the inability to conduct continuous observations at the same location. Sensor deployment density may be as low as one point source sample per 100 square kilometers, resulting in data with characteristics such as limited data volume, discrete spatial distribution, discontinuous time series, and high vertical sampling density [3,4]. Due to the inherent sparsity and discontinuity of data acquired from shipborne observations, data deviations caused by environmental anomalies or sensor malfunctions are difficult to effectively identify and correct. This limits the reliability and consistency verification of deep-sea environmental data to some extent, and increases the difficulty of achieving efficient anomaly detection and data correction. Therefore, reconstructing the distribution at the station scale from limited discrete point source observation data is the basis for understanding the evolution of the deep-sea environment and the key to effectively detecting and correcting anomalies [5].

In ocean sensor data, anomalous data primarily manifests as significant outliers caused by sensor failures or abrupt environmental changes, which are easily identifiable by traditional rule-based or statistical methods. However, more challenging are the concealed anomalies that fall within reasonable value ranges but are inconsistent with the spatio-temporal correlations of the ocean and the physical laws of vertical stratification. These anomalous data, combined with the inherent data limitations of sparsity, significantly increase the difficulty of point-based reconstruction and consistency verification, and also limit the applicability of traditional grid-based methods. Existing anomaly detection and correction technologies for this type of data include: (1) Rule-based automated quality control, including thresholding, gradient checks, and consistency checks, etc. Its advantages are interpretability and ease of deployment. However, constrained by baseline environmental differences across ocean regions and characteristic differences between different water layers, its ability to identify non-stationary environments and concealed anomalies is limited. (2) Unsupervised detection based on statistics and machine learning, including methods like Isolation Forests [6], Support Vector Machines (SVM) [7], and Variational Autoencoders [8]. These approaches identify anomalies via reconstruction error and are suitable for multivariate data, but they typically ignore site topology and voyage structure. (3) Time series algorithms such as ARIMA and LSTM can detect anomalies. However, they lack cross-site spatiotemporal relationships and rely on complete, continuous time series data for modeling [9,10,11]. This makes it difficult to capture the complex spatiotemporal correlations between data points.

The shipborne observation network and the sensor data it generates essentially constitute a complex, non-grid, relation-based data structure. The connections between each deployment site need to consider not only geographical proximity and time periodicity, but also the unique vertical hierarchical structure of the ocean environment. This topological structure, composed of heterogeneous spatiotemporal relationships, naturally fits the modeling paradigm of graph neural networks. In recent years, graph neural networks (GNN) have been widely used for field reconstruction or joint distribution estimation by learning node embeddings of non-grid, relational data through message passing and using reconstruction errors or probability biases to identify anomalies [12,13]. To explicitly describe the dynamic evolution of environmental features and observation network topology, spatiotemporal graph neural networks (STGNN) combine graph convolution with temporal encoders and memory mechanisms such as temporal convolutional networks and Transformers. This combination enables a unified modeling of spatiotemporal dependencies [14]. Ye et al. developed a spatiotemporal model using a graph convolutional network, achieving high-precision forecasts of sea surface temperature and chlorophyll-a [15]. Ou et al. proposed a spatiotemporal graph neural network based on incremental learning, achieving accurate predictions of ocean parameters [16]. However, the fragmentation of sparse ocean sensor data in the spatiotemporal dimension makes it difficult for STGNN to construct an effective spatiotemporal graph in this scenario. Since homogeneous GNNs usually assume a single type of node and a single type of edge [17], it is difficult to simultaneously represent multiple relationships such as geographical proximity, temporal correlation, and deep layering. Heterogeneous Graph Neural Networks (HGNN) [18] simulate heterogeneous connections through a message passing mechanism with multiple types of edges and specific relationships. Significant results have been achieved in fields such as communication, transportation, and meteorology. Li et al. proposed a heterogeneous temporal graph reinforcement learning algorithm, which was successfully used to optimize the channel allocation of the maritime Internet of Things [19]. However, research reports on HGNN in the field of marine environmental monitoring are still relatively rare. In fact, the characteristics of HGNN are highly compatible with the requirements of DeepData observation network structure and deep-sea environmental quality control, and have broad application prospects.

In summary, existing methods for sparse ocean sensor data still have significant limitations and deserve further investigation, which is the motivation for this study. We propose DAHSGNN, a Depth-Aware Heterogeneous Spatiotemporal Graph Neural Network. The main contributions are as follows:

(1) For sparse ocean sensor data with discrete observation points and inability to construct continuous observation time series, we propose a novel graph construction method based on a sliding window along the depth axis. This method leverages the strong correlation and continuity of ocean environmental parameters in the vertical dimension, combining fragmented spatiotemporal observations with the vertical physical structure, forming a graph topology that can capture vertical physical processes.
(2) Owing to the ocean’s non-stationary stratification, environmental parameters exhibit different trend and morphological patterns in different water layers. To capture these intra-layer patterns, we devised depth-aware hierarchical node feature engineering to effectively transform raw sensor data into structured graph nodes. We employ a Gaussian Hidden Markov Model (Gaussian HMM) to partition water layers and propose a weighted trend encoder guided by water-layer probabilities. A Transformer-based architecture independently encodes different water layers, and a cross-layer fusion mechanism captures inter-layer relationships. A Bidirectional Long Short Term Memory (BiLSTM)-based deep sequence encoder provides rich node features.
(3) The ocean environment exhibits complex phenomena involving relationships such as geographical proximity, temporal periodicity, water-layer stratification effects, and abrupt physical transitions [20]. Homogeneous graphs struggle to simultaneously represent these complex relationships. Therefore, we employ a HGNN to explicitly construct heterogeneous edges to accurately model the complex relationships between different observation stations.
(4) This method models various environmental variables based on the aforementioned heterogeneous graph structure and utilizes the reconstruction error of the heterogeneous graph autoencoder for anomaly detection and correction. This method further exhibits strong cross-variable transferability, achieving high reconstruction accuracy across diverse environmental variables while maintaining high sensitivity to anomalous data.

This method provides a scalable technical approach for modeling sparse ocean sensor data under shipborne survey conditions. Its efficient validation on multiple environmental parameters such as temperature, salinity, and turbidity in the International Seabed Authority (ISA) DeepData database demonstrates its generalization capability for discrete ocean observation scenarios.

## 2. Problem Description and Method

A non-grid spatial layout composed of ship trajectories and discrete sites is naturally suited for a graph representation. Traditional spatiotemporal methods rely on fixed grid-based adjacency and time series, making it difficult to uniformly represent the heterogeneous coupling relationships between geographic location, temporal evolution, and depth profiles in ocean data. While homogeneous graphs can attempt to model such interactions by designing complex relationship types, heterogeneous graphs offer a more efficient modeling paradigm. Considering the limitations of the data and the advantages of heterogeneous graphs, we reformulate the task of shipborne measurement anomaly detection and correction as a node-level physical quantity reconstruction problem on a heterogeneous graph, thereby converting discrete sensor observation data into a structured heterogeneous graph.

A heterogeneous graph [21], denoted as $G_{t}=(V_{t},E_{t},A,R)$, consists of an object set V and a link set E. A heterogeneous graph is also associated with a node type mapping function τ: V → A and a link type mapping function φ: E → R. A and R denote the sets of predefined node types and edge types, where |A| + |R| > 2. Among them, the node set $V_{t}$ consists of all the observation data points in the window. Considering the *homogeneous nature of shipborne survey data, all nodes are abstracted as a single type. Each node $v\in V_{t}$ corresponds to an observation point, some of which are shown in Table 1. The set $E_{t}$ contains edges of multiple relation types, and the set R defines all relation types. The graph topology is stored efficiently using an edge index dictionary, where each key-value pair records the sparse adjacency matrix M for a specific edge type. Each edge represents a particular physical or logical association, and edge weights are represented by weighted, typed adjacency matrices.

(1) $$M^{\left(r\right)}\in R^{N\times N}$$

(2) $$M_{ij}^{\left(r\right)}=\left\{\begin{array}{cc} w_{ij}^{\left(r\right)}, & (v_{i},v_{j})\in E_{r},\\ 0, & otherwise, \end{array}\right.$$

where $w_{ij}^{\left(r\right)}$ is the edge weight corresponding to the relationship type r.

**Figure 1:**
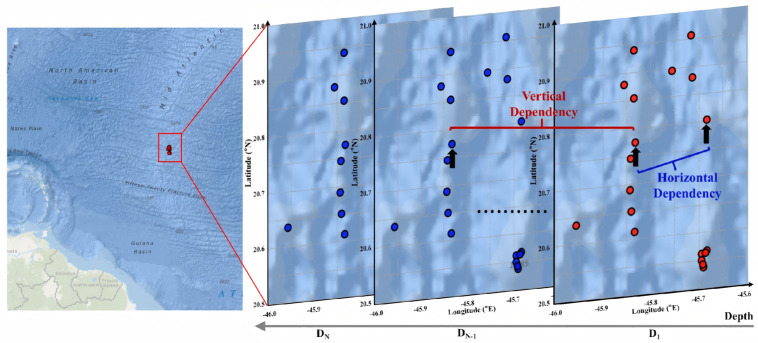
Geographic locations of shipborne CTD observation stations along the Mid−Atlantic Ridge. The red circles in the diagram represent some sampling sites, and the blue circles represent nodes at different depths within the same site.

We process a collection of oceanic profiles $P=\{P_{1},P_{2},\ldots ,P_{S}\}$ obtained at various times and locations, with S being the total number of profiles. As shown in Figure 1, all observation profiles share the same domain and timestream.

**Figure 2:**
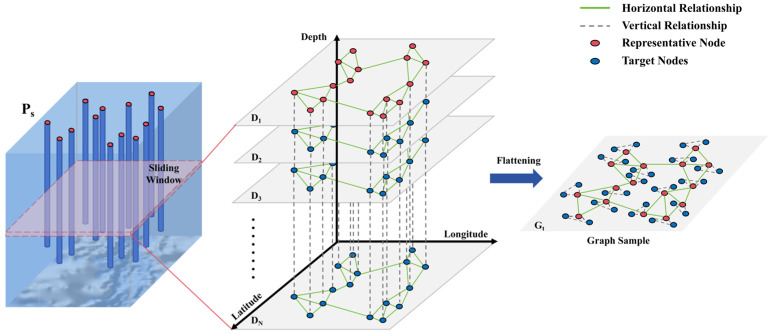
A schematic diagram of constructing a graph sample from consecutive depth slices within a window. It shows both lateral and vertical relationships between nodes.

To address the temporal discontinuities in shipborne sensor data and leverage local correlations in the vertical dimension, we propose a local graph construction method based on a depth sliding window. This method slides a fixed-length window along the depth direction of the vertical axis, aggregating discrete observation points within the window into a local graph sample, thereby capturing complex spatiotemporal-depth correlations. We use a sliding window of length N to slice the depth axis. For each window t, all observation points within the covered depth range are aggregated into a single graph to form the node set $v_{t}$. Under four relation criteria defined in R, we then generate the corresponding typed edge sets $M_{t}^{\left(r\right)}$ and weighted adjacency matrices $W_{t}^{\left(r\right)}$. This yields a graph sample $G_{t}$. By traversing all windows in sequence, we obtain a sequence of graphs $G=\{G_{1},G_{2},\ldots ,G_{T}\}$. Windowing partitions large-scale data into a set of size-controlled, relationally complete local heterogeneous graphs. This approach preserves the vertical structure and facilitates efficient batch training and inference, as illustrated in Figure 2. These heterogeneous graph samples are subsequently processed in batches into the heterogeneous graph autoencoder for training and inference.

For shipborne-acquired sensor data, we construct a heterogeneous graph-based reconstruction model $f_{\Phi }$, which integrates node features and the relationships between nodes in three dimensions: geospatial, temporal evolution, and vertical depth. Let $P_{t}\in R^{N_{t}}$ denote the vector of ground-truth physical quantities for all $N_{t}$ nodes in graph $G_{t}$. The reconstructed vector ${\hat{p}}_{t}$ is inferred by the model as ${\hat{p}}_{t}=f_{\Phi }(G_{t})$, where Φ denotes the learnable parameters. Because anomalous samples in the shipborne observations within DeepData are exceedingly rare, we adopt an unsupervised learning approach. The model parameters Φ are trained by minimizing a composite reconstruction loss function on a preprocessed graph sequence.

After training, for any observation node i, its anomaly score is defined by its reconstruction error with respect to $\left|{\hat{p}}_{i}-p_{i}\right|$. An adaptive threshold selection method based on the validation set is used to dynamically determine the optimal threshold by maximizing the F1-Score. If this error exceeds a threshold dynamically determined based on the reconstruction error distribution of all samples, the data point is considered to have deviated from its normal pattern and is assigned a higher anomaly probability.

## 3. DAHSGNN

The DAHSGNN method for sparse ocean sensor observations follows a multi-stage processing flow, comprising three core steps: (1) Depth-aware hierarchical node feature engineering: Extract the depth distribution pattern of the profile data of each station and generate node attribute vectors to represent the vertical distribution characteristics of the station at the corresponding depth. (2) Heterogeneous edge construction: Heterogeneous edges are constructed through domain knowledge and together with nodes form a heterogeneous graph. (3) Heterogeneous graph modeling: Feed the graph samples into a HGNN Autoencoder, which leverages the complex topology and node features to learn latent representations, thereby modeling geographical, temporal, and vertical dependencies.

### 3.1. Depth-Aware Hierarchical Feature Engineering

**Figure 3:**
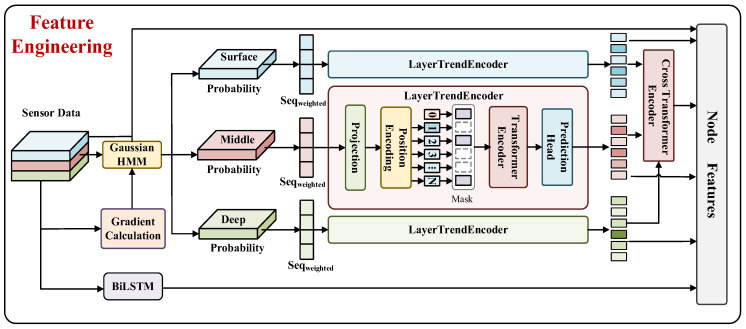
Depth−Aware Hierarchical Feature Engineering Schematic.

As shown in Figure 3, each node is associated with a feature vector rich in physical information, derived from the raw observations collected by the sensors. The vertical stratification of the ocean exhibits obvious heterogeneity. Different water layers have unique physical properties, and the water layers are mostly diffuse transition zones. In order to probabilistically divide the inherent complex vertical structure in the observation node data, we adopt the Gaussian HMM and use its ability to model the hidden state and its transition to explicitly characterize the water layer constraints [22]. In order to capture the intra-layer variation trend of the water layer, we use the sequence modeling capability of the Transformer architecture to generate a water layer weighted depth trend vector. In addition, a cross-layer fusion mechanism is designed to capture the interrelationship between water layers. A BiLSTM encoder is introduced to extract dynamic context features of local depth sequences [23].

#### 3.1.1. Water Layer Probability Division Based on Gaussian HMM

**Figure 4:**
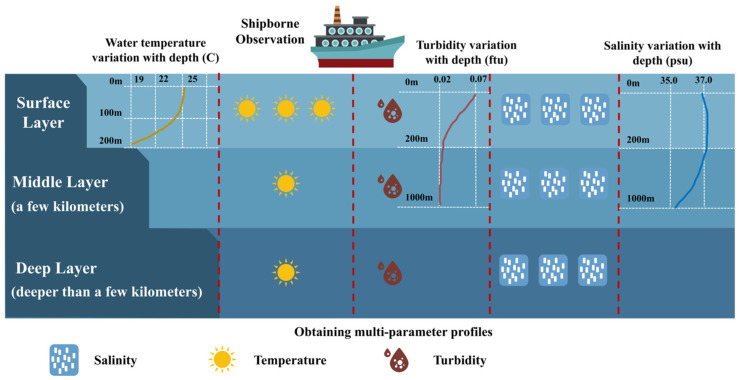
Mid−Atlantic Ridge Ocean Water Layer Division and Environmental Parameter Variations with Depth.

According to classical physical oceanography, the ocean’s vertical structure is commonly conceptualized as three layers with distinct physical properties [24], comprising shallow waters that are readily influenced by the atmosphere, a mid-layer that serves as an important buffer, and a vast, relatively stable deep layer [25]. Figure 4 shows the water column division in the Mid-Atlantic Ridge area and the changes in key environmental parameters with depth.

**Figure 5:**
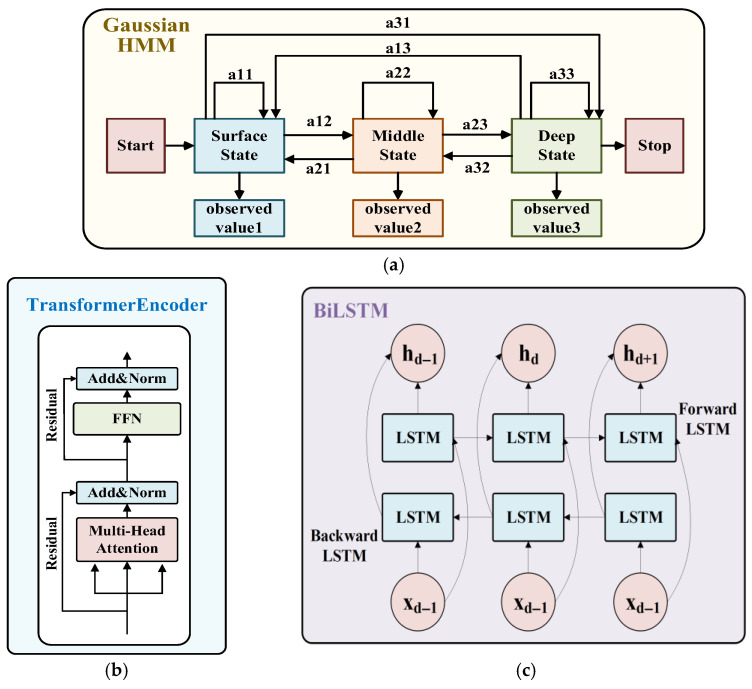
Network architectures for (**a**) Gaussian Hidden Markov Model, (**b**) Transformer, and (**c**) Bidirectional LSTM.

To accurately characterize the physical layered structure of the ocean, we employ the Gaussian HMM for probabilistic water-layer partitioning, as shown in Figure 5a. This model combines discrete state transition processes with continuous sensor observations, using a sequence of hidden states to model transitions between layers. The probability density function of the multivariate Gaussian distribution is used to process continuous physical quantities. The hidden state sequence $q_{t}\in S=\{s_{surf},s_{mid},s_{deep}\}$ corresponds to the three water layers, while the observation sequence $O=\{O_{1},O_{2},\ldots ,O_{T}\}$ consists of the multivariate feature vectors of the nodes. State transition probability matrix $A={\left[a_{ij}\right]}^{3\times 3}$:

(3) $$a_{ij}=P\left(q_{t+1}=s_{j}|q_{t}=s_{i}\right),\;\sum_{j=1}^{3}a_{ij}=1$$

The covariance matrices $\Sigma_{j}$ are learned from the data via the Baum-Welch algorithm [26]. Finally, using the trained model parameters, the posterior probability of belonging to each water layer is calculated for each observation point i:

(4) $$p_{i}={\left[P(q_{i}=s_{surf}|O),P(q_{i}=s_{mid}|O),P(q_{i}=s_{deep}|O)\right]}^{⊤}$$

This probability vector serves as the key representation of the vertical structure and is input into the subsequent trend encoding process.

#### 3.1.2. Water Layer Probability-Guided Trend Encoder

Different observation stations may exhibit similar environmental evolution trends due to driving factors such as ocean currents and climate patterns. Different water layers are influenced by physical, chemical, and biological processes, resulting in significant differences in their internal trends and morphological patterns. To effectively capture intralayer distribution patterns and correlate similar nodes, we propose a trend encoder.

The trend encoder learns a local dynamic trend vector $t_{i}$ for each site i. At its core is a self-attention mechanism guided by the water-layer probability $p_{i,l}(t)$ at depth index t. We employ a hierarchical Transformer architecture [27] that extracts specialized features for different water layers, comprising three parallel Transformer encoder layers, each dedicated to one layer, as illustrated in Figure 5b. When computing self-attention within layer l, attention is modulated by the joint probability that both points belong to the same layer:

(5) $$\alpha_{t,u}^{\prime (l)}=\alpha_{t,u}^{\left(l\right)}\cdot p_{i,l}(t)\cdot p_{i,l}(u)$$

where $\alpha_{t,u}^{\left(l\right)}$ is the standard self-attention score between two depth points t and u within the layer-l sequence. This modulation prioritizes interactions between points that are confidently assigned to layer l, while suppressing spurious cross-layer interactions.

We replace standard self-attention with joint probabilistic modulation of attention to form the encoded representation $h_{i,l}(t)$ at position t. Then, we obtain the layer-specific trend vector through probabilistic weighted pooling:

(6) $$v_{i}^{\left(l\right)}=\frac{\sum_{t}w_{i,l}(t)\;h_{i,l}(t)}{\sum_{t}w_{i,l}(t)+\varepsilon }$$

where $w_{i,l}(t)$ are the CHMM-derived layer probabilities used as weights and ε is a small constant for numerical stability. This prioritizes processing high-confidence intra-layer interactions to generate $v_{i}^{\left(l\right)}$ that reflects the true intra-layer distribution pattern at site i. Each depth point is labeled with location encoding to achieve ordered depth-related trend learning.

The outputs of each Transformer layer are stacked to construct a hierarchical feature sequence, which is then input into a cross-layer Transformer to model the interdependencies between different water layers [28]. This module is based on the standard Transformer encoder architecture, globally capturing the relationships between trend features of different water layers through a multi-head self-attention mechanism, and enhancing the non-linear expressive power of the features through a feedforward neural network. Finally, average pooling is applied to the output sequence of the cross-layer Transformer to generate a unified cross-layer trend vector:

(7) $$v_{i}^{\text{cross}}=\frac{1}{L}\sum_{l=1}^{L}{\left({\text{Transformer}}_{\text{cross}}([v_{i}^{\left(1\right)},\ldots ,v_{i}^{\left(L\right)}])\right)}_{l}$$

where L is the number of layers. ${\text{Transformer}}_{\text{cross}}$ models the dependencies between different water layers.

Finally, to form the node-level integrated trend vector, we apply a probability gate to each layer’s contribution and then concatenate the gated layer-wise vectors with the cross-layer vector:

(8) $$t_{i}=concat\left(g_{i}(1)\cdot v_{i}^{\left(1\right)},...,g_{i}(L)\cdot v_{i}^{\left(L\right)},v_{i}^{cross}\right)$$

With the gate

(9) $$g_{i}(l)=\left\{\begin{array}{ll} p_{i,l}(t_{i}), & if\;p_{i,l}(t_{i})>\tau ,\\ 0, & \text{otherwise} \end{array}\right.$$

The threshold τ is a dynamic threshold used to filter significant water layers. τ is adaptively generated by the dominant confidence of the water layer probability distribution of node i:

(10) $$\tau_{i}=\frac{1}{2}\underset{l}{\text{max}}(p_{i,l})$$

The physical information of a water layer is considered significant only when the probability $p_{i,l}$ of a certain water layer is higher than the threshold $\tau_{i}$, which is dynamically scaled by the dominant layer confidence. This design achieves: (1) Strict noise reduction in regions with clearly defined water layer stratification: When the confidence of the dominant water layer is high, the threshold is increased to retain significant water layer information. (2) Fusion of multiple physical layers in water layer transition regions: When the confidence of the dominant layer is low, the threshold is decreased to accommodate information from other water layers. This gating mechanism ensures that nodes only carry trend information of the layer to which they may belong, while preserving layer-specific structure and cross-layer context through splicing.

Through layer-by-layer specialization and cross-layer fusion, an integrated trend vector $t_{i}$ that incorporates cross-layer relationship information is ultimately generated. This integrated vector provides a high-dimensional representation of the physical state relevant to the water column at the node’s location and encodes information at two levels: (1) Positional Information: Positional encoding tags each depth point with its relative position within its inferred primary water layer. This enables the model to discern orderly depth-dependent trends in physical quantities. (2) Vertical structural information: It captures whether the intra-layer distribution of physical quantities is uniformly mixed, exhibits a gradual linear variation, or contains local extrema. This is used to characterize the inter-layer associations and transitional features among different water layers.

In this way, the model can identify spatially distant nodes that share similar structural characteristics, enabling the establishment of long-range dependencies based on physical properties.

#### 3.1.3. BiLSTM Deep Sequence Encoder

The profiler equipped with multi-parameter sensors performs in situ high-frequency sampling during the vertical descent process, so that the acquired ocean profile data shows extremely high data density, continuity and local dynamic changes in the vertical direction. When processing graph samples, nodes at the same site consisting of densely sampled points are sorted by depth, forming a depth sequence $X=(x_{1},x_{2},...,x_{N})$. Adjacent nodes in a depth sequence have close physical connections and dynamic interactions, and this dependency is bidirectional. The state of the upper water layer affects the lower layer, while processes in the lower layer, such as upwelling and heat conduction, also have feedback effects on the upper layer. To capture the continuity and local dynamics inherent in high-resolution vertical sampling, we use a bidirectional long short-term memory (BiLSTM) network as a deep sequence encoder. This encoder is used to extract the continuity and dynamic trend features of these local, depth-ordered sequences in the graph samples. Figure 5c shows the basic framework of BiLSTM. The BiLSTM consists of a forward LSTM and a backward LSTM. The forward LSTM processes the sequence from shallow to deep (d = 1 → N), recursively updating its hidden state. Conversely, the backward LSTM processes the sequence from deep to shallow (d = N → 1). Both propagate information based on the current input and the hidden states of adjacent layers. This mechanism enables the model, when generating features for any node at depth d, to simultaneously consider information from the shallower and deeper waters, thereby constructing a more complete local vertical environmental context.

Finally, the hidden states of the forward and backward LSTM layers at the current depth are concatenated to obtain a vector output that integrates the bidirectional context information of the sequence. We define this as the depth-sequence feature $h_{lstm}$.

(11) $$h_{lstm,d}=\left[\vec{h_{d}};\overset{\leftarrow }{h_{d}}\right]=\left[{LSTM}_{fwd}(x_{d},\vec{h_{d-1}});{LSTM}_{bwd}(x_{d},\overset{\leftarrow }{h_{d+1}})\right]$$

The extracted deep sequence features $h_{lstm}$ are concatenated with the original features of the node and the additional feature information obtained through feature engineering, forming the final node features $h_{i}^{\left(0\right)}$ that are input to the heterogeneous graph neural network.

### 3.2. Physical Information-Guided Heterogeneous Edge Construction

Considering the complex interactions within marine systems, we construct four types of heterogeneous edges based on geographic-distance, temporal, vertical, and trend similarities between nodes:

(1) Geographic Distance Edge: It aims to capture the spatial correlation of the ocean in the quasi-horizontal plane.
  (12) $$w_{ij}^{\text{Distance}}=e^{-\frac{dist({pos}_{i},\;{pos}_{j})}{\sigma^{2}}}$$
  where ${pos}_{i}$ is the geographical location (latitude and longitude) of sensor node i, dist( , ) is the Haversine distance between two points [29], and σ is the bandwidth parameter of the Gaussian kernel, controlling the rate of decay in similarity with distance [30].
(2) Temporal Edge: The physical state of marine environmental variables often exhibits seasonal and diurnal variations. Nodes sharing temporal similarities likely exhibit analogous ocean states. The weight is based on the cosine similarity between temporal feature vectors:
  (13) $$w_{ij}^{\text{time}}=\cos(f_{i}^{t},\;f_{j}^{t})$$
  where $f_{i}^{t}$ and $f_{j}^{t}$ are the temporal feature vectors of nodes i and j, respectively.
(3) Vertical Edge: By connecting pairs of points in the same profile with adjacent depths, similar measured environmental parameter values, and similar local curve shapes, a vertical edge is established to reflect the physical continuity and discontinuities of the water body. Its weight is a linear combination of multiple physical factors:
  (14) $$w_{ij}^{ver}=\frac{w_{dist}\cdot Sim(d_{i},d_{j};C_{1})+w_{sl}\cdot Sim(s_{i},s_{j};C_{2})+w_{grad}\cdot \frac{1}{2}\left(Sim(g_{i},g_{j};C_{3})+Sim(g_{i}\prime \prime ,g_{i}\prime \prime ;C_{4})\right)}{w_{dist}+w_{sl}+w_{grad}}$$
  where $Sim(a,b;\sigma )=e^{-|a-b|/\sigma }$ is an exponential decay function. $d_{i}$ and $s_{i}$ represent the depth and measured environmental parameter value of node i, $g_{i}$ and $g_{i}\prime \prime$ represent the gradient and second-order gradient of node i, $w_{*}$ is a hyperparameter tuned on the validation set, and $C_{*}$ is a scale parameter.
(4) Trend Edge: By calculating the similarity of trend vectors, similar oceanographic characteristics and generation mechanisms between sensor data from different observation sites can be captured:
  (15) $$w_{ij}^{trend}=\cos(t_{i},t_{j})$$
  $t_{i}$ is the comprehensive trend vector obtained by the Transformer encoder.

This study employs the KNN algorithm [31] to balance similarity capture and computational efficiency for four types of heterogeneous edges. This strategy may include a small number of edges with low connection strength during the selection process, but the subsequent neighborhood aggregation mechanism automatically weakens their contribution. Simultaneously, KNN may also miss a few highly similar nodes, but the selected topological associations cover the main dependencies, sufficient to support high-precision reconstruction of node physical quantities.

When constructing geographic distance edges and temporal edges, different depth nodes at the same site have the same geographical location and sampling time, resulting in identical edge weight calculations. To reduce redundant computations during edge construction, we use the shallowest depth sampling point of the site within the sliding window as the representative node. Only the weights between the representative nodes are calculated, and as shown in Figure 2, these weights are copied to other depth points of the site to obtain the final edge weights.

### 3.3. Heterogeneous Graph-Based Autoencoder Model

**Figure 6:**
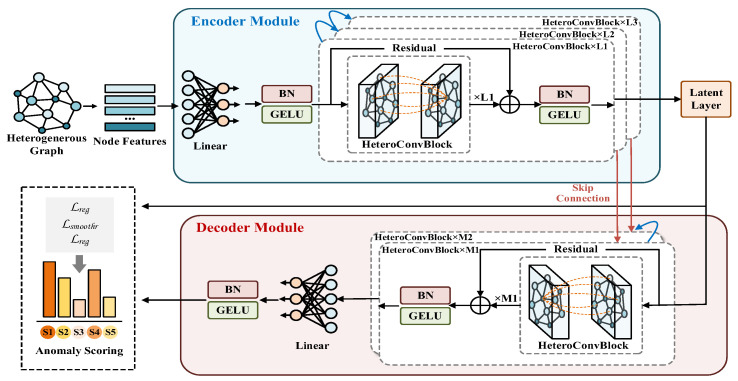
Heterogeneous Graph Autoencoder.

After the heterogeneous graph is constructed, the data is input into the heterogeneous graph autoencoder for anomaly detection. Figure 6 shows its detailed architecture. The core task of this model is to learn the inherent patterns and intrinsic structures of normal ocean profile data under high-dimensional spatiotemporal relationships. The encoder compresses the complex graph signal into a low-dimensional latent representation, and the decoder reconstructs the original signal. Since the model learns the normal data distribution pattern during training, the inherent abnormal structure of anomalous data leads to a high reconstruction error. Anomaly detection is achieved based on the reconstruction error, and data correction is performed based on the reconstructed values. The entire process, from graph sequence input, deep processing of node features and edge information, to the final output of reconstructed physical quantity values, is a continuous process.

Each node feature passes through an input projection module. This module consists of a linear layer, a batch normalization layer, and a GELU activation function. The input features are mapped to a higher-dimensional latent space, providing a more expressive initial representation for subsequent graph convolution operations.

**Figure 7:**
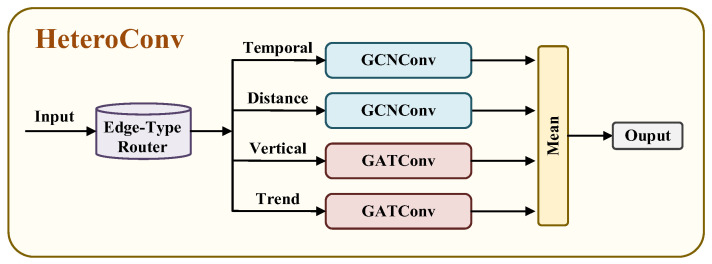
HeteroConv internal structure.

The data then flows through a core network consisting of multiple layers of heterogeneous graph convolution blocks. The core of each convolution block is the HeteroConv layer, which uses a dedicated message passing mechanism for edges $E_{type}$ with different physical meanings in the graph. For each type of relation, the model collects information from the neighboring nodes of the central node and aggregates it into a single message. As shown in Figure 7, appropriate graph convolution operators are selected based on the characteristics of different relations. To characterize the complex and non-uniform interactions within and between different depth layers, we employ a graph attention network (GATConv) on vertical and cross-layer trend edges. Its self-attention mechanism can adaptively learn and assign weights based on the characteristics of neighboring depth nodes, thereby highlighting key interactions and suppressing secondary connections in complex ocean environments, and improving the ability to model intra-layer and cross-layer dependencies. Geographic distance edges connect nodes in neighboring spatial locations, while temporal edges connect nodes deployed at different times. These two heterogeneous edges typically exhibit relatively smooth, region-dependent characteristics. Therefore, graph convolutional networks (GCNConv) [32] with higher computational efficiency are chosen to effectively capture relatively uniform and stable association patterns within geographical or temporal neighborhoods.

After aggregating neighbor information for each relationship type, the HeteroConv layer merges information from different relationship types into a unified neighborhood information through mean pooling. The forward propagation of layer l can be formalized as:

(16) $$h_{i}^{\left(l\right)}=GELU\left(BN\left(\frac{1}{\left|R\right|}\sum_{r\in R}AGG_{r}^{\left(l\right)}\left\{h_{j}^{\left(l-1\right)}\cdot w_{ij}^{\left(r\right)}|j\in N_{r}(i)\right\}+W_{res}h_{i}^{\left(l-1\right)}\right)\right)$$

$h_{i}^{\left(l\right)}$ represents the feature representation of node i at layer l. R represents the relation type of all edges. $AGG_{r}^{\left(l\right)}$ represents the aggregation function of layer l for relation r. $w_{ij}^{\left(r\right)}$ is the edge weight between nodes i and j under relation r. $h_{j}^{\left(l-1\right)}\cdot w_{ij}^{\left(r\right)}$ represents the weighted node features. The edge weights serve as physical reliability indicators, and the features of neighboring nodes with strong associations are given greater weight when passed to the central node i. $N_{r}(i)$ is the set of neighbors of node i under relation r, and $W_{res}$ is the learnable weight matrix of residual connections.

To ensure effective training of deep networks and prevent overfitting, batch normalization, GELU activation function, and dropout are integrated after each HeteroConv convolutional module. At the same time, residual connections [33] are introduced to combine neighborhood information with the original features of nodes to generate new representations of nodes and alleviate the gradient vanishing problem. After feature extraction and fusion through multi-layer graph convolution, the last layer of the encoder maps the high-dimensional representation of nodes to a low-dimensional latent space, generating the final latent representation matrix $Z\in R^{N\times d_{z}}$. This vector is the encoder’s final abstract representation of the input graph information.

The decoder takes the latent representation Z as input and restores the dimension of the node representation layer by layer through a stack of HeteroConv modules with the same structure but the reverse order of the hidden layer dimensions. However, it is difficult to recover all the details using only low-dimensional Z vectors. To address this, skip connections are introduced, directly passing the output feature maps of the corresponding layers in the encoder to the decoder, compensating for the details and high-frequency information lost due to information compression during encoding. Finally, at the end of the decoding network, the decoded high-dimensional node features are mapped back to a single-dimensional physical quantity through an output projection consisting of a linear layer, batch normalization, and GELU activation.

The model is trained in an unsupervised manner on a training set containing only normal samples. The goal is to optimize the parameters by minimizing a composite loss function, which consists of three parts:

(1) The mean square error (MSE) is used as the core reconstruction loss function:
  (17) $$L_{rec}=\frac{1}{N}\sum_{i=1}^{N}({\hat{s}}_{i}-s_{i}{)}^{2}$$
  where ${\hat{s}}_{i}$ is the reconstructed physical quantity value of node i, $s_{i}$ is the corresponding true physical quantity value, and N is the total number of nodes in the batch.
(2) Regularization loss for regulating the latent space:
  (18) $$L_{reg}=\frac{1}{N}\sum_{i=1}^{N}\|z_{i}{\|}_{2}$$
  where $z_{i}$ is the representation vector of node i in the latent space.
(3) Smoothness loss based on physical prior:
  (19) $$L_{smooth}=\frac{1}{M-1}\sum_{j=1}^{M-1}\left|s_{d_{j+1}}-s_{d_{j}}\right|$$
  where $s_{d_{j}}$ represents the reconstructed value of the physical quantity at depth $d_{j}$, and M is the number of nodes sorted by depth.

## 4. Data and Preprocessing

### 4.1. Dataset Description

**Figure 8:**
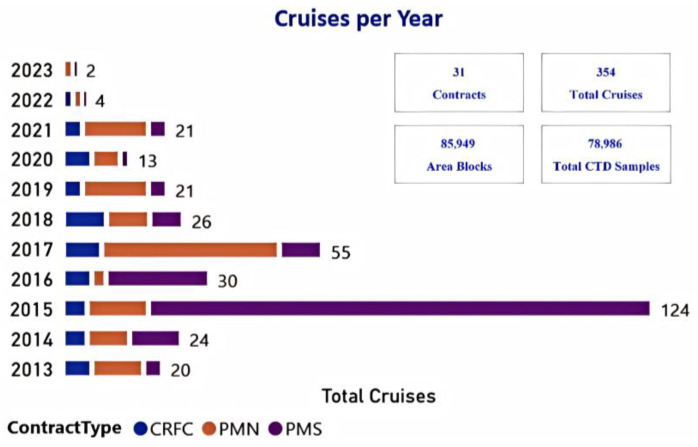
Statistics of cruise observation data within the DeepData database.

The ocean profile sensor data used in this study were obtained from the DeepData database established by the ISA. The database is dedicated to collecting data generated from activities related to ‘The Area’. It aggregates ocean environmental survey data from multiple ‘The Area’ worldwide, including the Mid-Atlantic Ridge, the Central Indian Ocean, and the Clarion-Clipperton Fault Zone. Figure 8 shows the shipborne observation data acquired from “The Area,” spanning from 2001 to 2023, encompassing 354 expeditions and covering over 80,000 Area Blocks. These data, primarily from high-density sampling by the shipborne multi-parameter profiler during the voyage, are characterized by being sparse, non-gridded, and having a high vertical sampling density. Based on the high degree of fit between its data characteristics and the research questions of this study, we used data from the DeepData database covering the period of 2012 to 2015, the period with the most observation cruises on the Mid-Atlantic Ridge, to verify the effectiveness of the proposed method. The dataset contains temperature, salinity, and turbidity profile data collected at specific stations in the region.

### 4.2. Data Preprocessing

This study is dedicated to reconstructing the correct distribution of site-level sensor data. However, due to environmental conditions or sensor failures, outliers will inevitably appear. If left unprocessed, they will affect the accuracy of model reconstruction training. Considering the existence of multiple types of anomalies in ocean data, a single anomaly detection method often fails to achieve ideal results. The Z-score method can quickly filter out single-dimensional extreme anomalies, but is insensitive to local anomalies [34]. Isolation forest can identify more subtle multidimensional structural anomalies, but may miss more discrete anomalies. Therefore, we combine Z-score and isolation forest to compute a weighted sum of anomaly scores. This produces a cleaner training dataset, which enables the model to learn the normal pattern of the data. The dataset is divided into training, validation, and test sets according to station ID, with a ratio of 6:2:2. To construct a quantifiable evaluation test environment, we manually injected labeled outlier data points into the validation and test sets. Based on the time-series data outlier injection method in reference [35], we simplified some techniques. Based on the statistical characteristics of the preprocessed data, we selected data points accounting for approximately 0.5% of the total data to inject outliers. The outlier injection location was determined by random sampling. Since the data characteristics change with depth, we calculated the standard deviation σ of the data within the sliding window centered on the outlier injection point as the noise intensity. Based on this, we set the offset between 2σ and 5σ to effectively simulate significant outliers and hidden anomalies. All injections were labeled with outlier tags for the calculation of evaluation metrics.

## 5. Experiment

### 5.1. Experimental Environment and Parameter Configuration

Model training and all experiments were performed using Python 3.12.11 and Torch 2.7.0 + cu12.8. The graphics card model was (NVIDIA GeForce RTX5070Ti, 24 GB, NVIDIA Corporation, Santa Clara, CA, USA). Table 2 lists the search range and considered variants for the model hyperparameters. Detailed parameters were tested using a grid search method, and the optimal hyperparameters were ultimately selected based on the validation set.

### 5.2. Evaluation Criteria

We evaluate the reconstruction performance of the proposed DAHSGNN method from three perspectives. The mean absolute error (MAE), root mean square error (RMSE), and mean absolute percentage error (MAPE) are used to quantify the reconstruction accuracy. We used precision, recall, F1 score, and precision-recall curve (PRC) to comprehensively evaluate the performance of the DAHSGNN model [36]. For the imbalanced characteristics of sparse ocean sensor data, the F1 score more effectively reflects the overall effectiveness of the model in balancing precision and recall. The PRC is a tool for evaluating model performance particularly suitable for imbalanced datasets. The average precision (AP) is an approximation of the area under the PRC, which comprehensively reflects the average performance of the model under all thresholds. Its formula is as follows:

(20) $$MAE=\frac{1}{n}\sum_{i=1}^{n}\left|y_{i}-{\hat{y}}_{i}\right|$$

(21) $$RMSE=\sqrt{\frac{1}{n}{\sum_{i=1}^{n}\left(y_{i}-{\hat{y}}_{i}\right)}^{2}}$$

(22) $$MAPE=\frac{100\%}{n}\sum_{i=1}^{n}\frac{\left|y_{i}-{\hat{y}}_{i}\right|}{y_{i}}$$

(23) $$A=\frac{TP+TN}{TP+TN+FP+FN}$$

(24) $$P=\frac{TP}{TP+FP}$$

(25) $$R=\frac{TP}{TP+FN}$$

(26) $$F1=\frac{2TP}{2TP+FN+FP}$$

(27) $$AP=\int_{0}^{1}P(r)\;dr$$

The above indicators can comprehensively reflect the performance of the model and help evaluate the applicability of the model in different scenarios.

### 5.3. Experimental Results and Analysis

To evaluate the contribution of each module in the model and verify its effectiveness, we conducted ablation experiments. While maintaining consistent training parameters, we analyzed the anomaly detection performance of DHASGNN under different module combinations. The results are shown in Table 3. The results indicate that the recall rate of the base model is relatively low, suggesting that the original features are insufficient to fully characterize the key features of ocean vertical stratification. After introducing TrendEncoder, the recall rate for salinity and turbidity data improved slightly, but the performance for water temperature data decreased. This difference stems from the physical characteristics of the environmental variables themselves. The vertical distribution of salinity and turbidity is usually dominated by diffusion and sedimentation, resulting in relatively gentle changes. Water temperature often exhibits more significant and non-linear gradient changes in the vertical direction, as shown in Figure 5. The self-attention mechanism of TrendEncoder is effective in capturing steady trends, but its ability to represent drastic gradient changes is limited. With the gradual introduction of Gaussian HMM and CrossEncoder, the model achieves explicit modeling of water layer state transitions and effective capture of inter-layer correlations, significantly improving model performance. This verifies the necessity of explicit stratification modeling for complex vertical structures. The BiLSTM module enhances the representation of local dynamic processes inherent in high-resolution vertical sampling by modeling bidirectional dependencies between adjacent depth nodes. Experiments demonstrate that the DAHSGNN model, integrating all modules, plays a crucial role in capturing complex spatiotemporal heterogeneous correlations. DAHSGNN achieves a superior balance between significantly improving recall and maintaining high accuracy, thus realizing a better anomaly detection performance.

**Figure 9:**
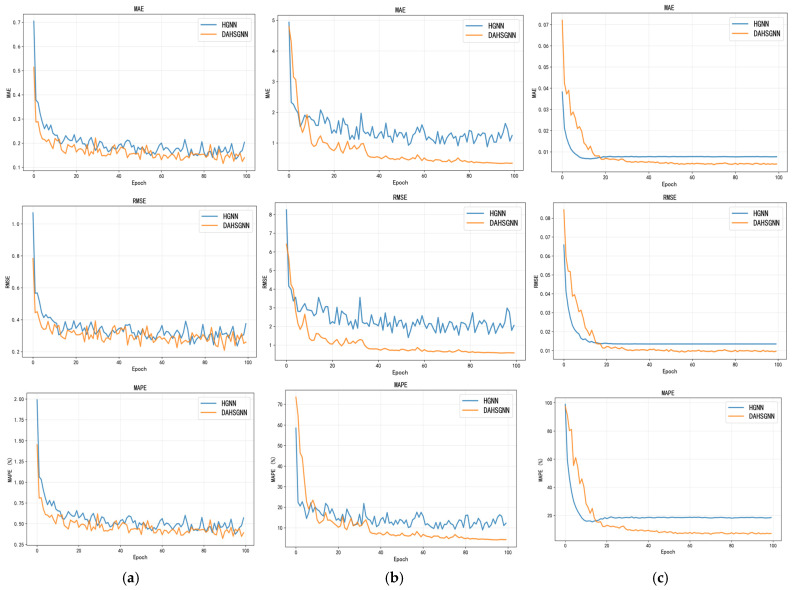
Comparison of model reconstruction performance for (**a**) salinity, (**b**) water temperature, and (**c**) turbidity.

Figure 9 compares the MAE, RMSE and MAPE performance metrics between DAHSGNN and baseline HGNN models over 100 training epochs. Results show DAHSGNN achieves significantly superior training performance across all environmental variables. This proves that DAHSGNN can accurately reconstruct normal patterns, laying a reliable foundation for anomaly detection and subsequent data correction based on high reconstruction errors.

**Figure 10:**
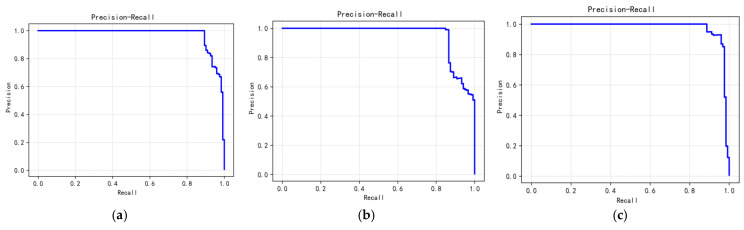
PRC of (**a**) salinity, (**b**) water temperature and (**c**) turbidity.

Figure 10 shows PRC for DAHSGNN on three environmental variables: salinity (AP = 0.97), water temperature (AP = 0.949), and turbidity (AP = 0.976). These high AP values demonstrate the model’s effectiveness in identifying anomalies through reconstruction error.

To comprehensively evaluate the performance of DAHSGNN, we conducted comparative experiments on the same dataset, including internal component replacement and end-to-end model comparisons. In the internal component replacement experiment, we replaced the joint module responsible for water layer partitioning and sequence modeling in DAHSGNN with the Mamba state-space model for comparison [37]. We also replaced the heterogeneous autoencoder in the original model with a Transformer to further explore the impact of different modules on performance. In the end-to-end model comparison, we compared and analyzed DAHSGNN with Convolutional Neural Network (CNN) [38], Graph Neural Network Based on Hierarchical Spatiotemporal Dependency Learning (HSDGNN) [39] and iTransformer model [40] which is suitable for multidimensional time series modeling. Table 4 shows the comparison results of anomaly detection performance of each model under different environmental parameters. The analysis results show that, compared with the Mamba state-space model, DAHSGNN’s explicit modeling of ocean physical stratification effectively improves the representation capability of sparse ocean sensor data. Replacing the heterogeneous graph autoencoder with a Transformer resulted in a performance decrease, demonstrating the advantages of heterogeneous graphs in fusing multi-source heterogeneous ocean data. Replacing the heterogeneous graph autoencoder with a Transformer highlights the advantages of heterogeneous graphs in fusing multi-source heterogeneous ocean data. Compared with the general sequence modeling framework iTransformer, DAHSGNN achieves better results in capturing spatiotemporal dependencies. Meanwhile, the overall performance of DAHSGNN also surpasses that of the CNN and HSDGNN benchmark models. This confirms the necessity of the domain knowledge-guided architecture design in this paper.

## 6. Conclusions

This paper aims to address the challenges posed by the inherent spatial discreteness and temporal discontinuity of sparse ocean sensor data. We propose a deep-aware heterogeneous spatiotemporal graph neural network (DAHSGNN) for anomaly detection and correction in sparse ocean sensor data. This method, based on sensor data, segments water layers using a Gaussian HMM and extracts multidimensional features using Transformer-based water layer trend encoding and BiLSTM modeling. It captures the complex dependencies of ocean sensor data in terms of vertical profile, geographic distribution, and temporal dynamics by constructing a depth-sensing heterogeneous map, thereby processing dense depth sampling sequences. Experiments demonstrate that, for multiple environmental variables (salinity, water temperature, turbidity), DAHSGNN outperforms traditional homogeneous graph models and benchmark heterogeneous graph models, validating its efficacy in sparse ocean sensor data scenarios.

Future research will focus on investigating the coupling effects among multiple environmental variables to achieve multivariate joint modeling and adaptive water layer states. It will also explore innovative methods for adaptively delineating water layer boundaries through a fusion strategy of physical constraints and data-driven approaches. We will also expand the reconstruction of site-scale data to encompass the entire “Area” environmental field. We will also promote the evolution of models towards lightweight and scalable models to meet the needs of larger-scale, higher-resolution marine environmental monitoring.

In summary, this study provides an effective solution to the problem of anomaly detection and correction in sparse ocean sensor data, and the solution has been validated in the ISA DeepData database. Through continuous improvement and multidisciplinary integration, this method is expected to provide a scalable technical path for data quality optimization in the field of deep-sea environmental monitoring.

## Figures and Tables

**Table 1:** Geographic coordinates and water depths measured at selected shipborne survey stations in the Mid-Atlantic Ridge region.

Location	35-1	35-13	35-107	36-58	36-83	36-212	37-103	37-154	37-169	37-190	37-199	37-209
Longitude (◦)	−45.63	−45.79	−45.74	−45.87	−45.76	−46.61	−46.36	−46.45	−46.56	−46.39	−46.41	−46.68
Latitude (◦)	20.05	20.69	20.15	19.88	20.05	17.93	17.14	16.74	16.73	17.15	17.12	17.82
Depth (m)	1950	2810	2402	2861	2493	3131	1510	4618	2762	1904	3016	3694

**Table 2:** Hyperparameters of the model.

Parameters	Value or Type	Search Range/Variants Considered
Epochs	150	[50, 100, 150, 200, 300]
Batch Size	16	[4, 8, 16, 32, 64]
Optimizer	Adam	[SGD, Adam]
Window Size	20	[15, 20, 30, 40, 50]
Step Size	8	[3, 5, 8, 10, 15]
Initial Learning Rate	0.005	[0.0001, 0.0005, 0.001, 0.005, 0.01]
Dropout	0.1	[0, 0.1, 0.2, 0.3, 0.4]
Early Stopping	30	[10, 15, 20, 30, 40]
Reconstruction weight	0.5	[0.3, 0.5, 0.7]
Smoothness weight	0.05	[0.01, 0.05, 0.1, 0.3]
Latent weight	0.001	[0.001, 0.01, 0.05]

**Table 3:** Ablation experiments.

	Gaussian HMM	TrendEncoder	CrossEncoder	BiLSTM	Param	Acc (%)	Pre (%)	Rec (%)	F1-Score(%)
1	×	×	×	×	Sal	99.80	100	80.95	89.47
					Tem	99.87	100	80.34	89.10
					Tur	99.77	100	79.31	88.46
2	×	√	×	×	Sal	99.90	100	90.48	95.00
					Tem	99.83	83.17	93.33	87.96
					Tur	99.77	100	79.31	88.46
3	×	√	√	×	Sal	99.90	95.24	95.24	95.24
					Tem	99.85	87.23	91.11	89.13
					Tur	99.77	100	79.31	88.46
4	√	√	×	×	Sal	99.98	74.42	96.97	84.21
					Tem	99.91	93.33	93.33	93.33
					Tur	99.97	100	95.9	97.91
5	√	√	√	×	Sal	99.98	96.97	96.97	96.97
					Tem	99.96	100	94.44	97.14
					Tur	99.97	100	95.0	97.44
6	×	×	×	√	Sal	99.8	79.1	99.1	87.98
					Tem	99.8	80	93.33	86.15
					Tur	99.8	79.1	99.1	87.98
7	×	√	×	√	Sal	99.92	100	87.8	93.50
					Tem	99.83	83.84	92.22	87.83
					Tur	99.92	100	87.8	93.50
8	×	√	√	√	Sal	99.94	100	90.4	94.96
					Tem	99.84	84.69	92.22	88.29
					Tur	99.94	100	90.4	94.96
9	√	√	×	√	Sal	99.99	99.14	100	99.57
					Tem	99.96	100	93.33	96.55
					Tur	99.99	99.14	100	99.57
10	√	√	√	√	Sal	99.99	99.14	100	99.57
					Tem	99.97	100	95.56	97.73
					Tur	99.99	99.14	100	99.57

Note: √ represents the use of this module, while × indicates that the module was not used.

**Table 4:** Comparison experiments with other models on multiple environmental parameters.

Model	Param	Acc (%)	Pre (%)	Rec (%)	F1-Score (%)
Mamba	Sal	99.97	100	95.56	97.73
	Tem	99.81	98.78	74.31	84.82
	Tur	99.83	100	76.61	86.76
Transformer	Sal	99.98	78.38	87.88	82.86
	Tem	99.85	98.61	78.89	87.65
	Tur	99.98	80.71	86.43	83.47
iTransformer	Sal	99.98	85.08	89.17	87.08
	Tem	99.98	84.60	92.17	88.22
	Tur	99.72	72.29	82.09	76.88
CNN	Sal	99.42	55.09	75.41	63.67
	Tem	99.86	90.37	86.52	88.40
	Tur	99.67	67.26	84.72	74.99
HSDGNN	Sal	99.99	97.26	89.17	93.04
	Tem	99.99	96.43	86.52	91.21
	Tur	99.97	97.43	91.55	94.40
DAHSGNN	Sal	99.99	99.14	100	99.57
	Tem	99.97	100	95.56	97.73
	Tur	99.99	99.14	100	99.57

## Data Availability

The original contributions presented in this study are included in the article. Further inquiries can be directed to the corresponding author.
